# Predicting handgrip power of young adult population among major ethnic groups of Sabah: a multivariate analysis

**DOI:** 10.1186/s40101-022-00297-x

**Published:** 2022-06-04

**Authors:** M Tanveer Hossain Parash, Hasanur Bin Khazri, Zainal Arifin Mustapha, Sadia Choudhury Shimmi

**Affiliations:** 1 Department of Biomedical Sciences, Faculty of Medicine and Health Sciences, Universiti Malaysia Sabah, Kota Kinabalu, Sabah Malaysia; 2 Borneo Medical and Health Research Centre, Faculty of Medicine and Health Sciences, Universiti Malaysia Sabah, Kota Kinabalu, Sabah Malaysia; 3 Department of Medical Education, Faculty of Medicine and Health Sciences, Universiti Malaysia Sabah, Kota Kinabalu, Sabah Malaysia

**Keywords:** Hand strength, Handbreadth, Gender, Ethnicity, Linear models, Sabah, Malaysia

## Abstract

**Background:**

Handgrip power is an essential indicator of health, vital for grasping or gripping sports, and crucial for providing information related to work capacity. The present study investigated any linear relationship of handgrip power with hand anthropometric variables (hand length, handbreadth, middle finger length, second inter-crease length of the middle finger, and hand span), gender, and ethnicity in young adults of Sabah.

**Methods:**

In this cross-sectional study (from January 2020 to December 2021), the adult Sabahan population (18-25 years) was stratified into four ethnicities (KadazanDusun, Bajau, Malay, and Chinese) and was further stratified as males and females. Then, 46 subjects were randomly selected from each gender, and the ethnic group met the intended sample size. The hand dimensions were measured using a digital calliper, and the handgrip power was measured using a portable dynamometer. The relationship between the response variable and explanatory variables was analyzed at first through simple linear regression and then multiple linear regression. *R*^2^, adjusted *R*^2^, and standard errors of the estimates were used to compare different models. Statistical analyses were performed using IBM SPSS Statistics 27 and StatCrunch.

**Results:**

The study found a linear relationship between gender, height, hand length, handbreadth, hand span, middle finger length, and second inter-crease length of both hands with the corresponding hand’s grip power. The highest percentage (68% and 67%) of handgrip variability was demonstrated by the model predicting handgrip power for right-handed subjects, followed by the general models without stratifying based on hand dominance which was able to explain 63% and 64% of the variability of handgrip power. The study proposes the models for predicted right (RHGP) and left handgrip power (LHGP) of 18 to 25 years old adults from major ethnic groups of Sabah RHGP = − 18.972 − 8.704 Gender + 7.043 Right hand breadth and LHGP = − 11.621 − 9.389 Gender + 5.861 Left hand breadth respectively.

**Conclusion:**

The predicted handgrip power would be a key to selecting a better player or a better worker or assessing the prognosis of a disease or the wellbeing of a person. The study can be further expanded to all ethnicities and ages of people of Sabah or even Malaysia.

## Background

Handgrip power is an essential indicator of health, vital for grasping or gripping sports, and crucial for providing information related to work capacity [1–3]. Measurement of handgrip power is vital to tracking anyone’s development, aging, injury, rehabilitation, training, or therapeutic trials [4]. Researchers demonstrated a crucial relationship between handgrip power, forced expiratory volume in one second (FEV_1_), and forced vital capacity (FVC), which are significant predictors of pulmonary function [5]. Handgrip power is associated with several chronic diseases, cognitive decline, length of hospital stays, and mortality [6–9]. A cross-sectional study in the chronic phase after stroke demonstrated that handgrip power is strongly associated with arm muscle strength. Again, grip strength measurement is more accessible and less time-consuming than arm muscle strength measurements. The study suggested that grip power could represent muscle weakness of the entire upper extremity in the chronic phase after stroke [10].

Several sports that require gripping and engaging power, such as weightlifting, golf, hockey, tennis, mountain climbing, baseball, paddling, swimming, and wrestling, need ample handgrip power to optimize performance and prevent injury [11]. Several researchers found that between elite and non-elite young judo athletes [12], elite and non-elite American junior-aged men weightlifters [13], elite and amateur female Olympic wrestlers [14], female elite and recreational rock climbers and non-climbers [15], and division I hockey players and division III players [16], the superior demonstrated greater handgrip power than their fellows. However, some research yielded insufficient evidence to prove that elite players had more handgrip power than their counterparts [17–20].

The ergonomic hand tools are designed and selected based on handgrip power to ensure the safety of manual tasks [21]. The handgrip strength evaluates available muscular strength related to work capacity, and this information can be used for designing equipment, workstations, and tasks to fit the strength of specific populations. The purpose of appropriate work design principles during the design of tools and workstations that require grip strength is to minimize the potential injuries due to mismatches between job demands and the capacity of workers [3]. Again, a firm grip secures robust and steady shoulders, allowing one to maintain a stable position while focusing and absorbing the recoil while shooting. Researchers have found that a law enforcement officer with a firmer handgrip power demonstrated superior shooting performance [22].

From the literature review, it is evident that handgrip power has many practical applications. Currently, many instruments can be used to measure the handgrip power with minimal errors. However, plenty of publications supports one instrument over another, while measuring the handgrip power or strength is not that simple. The researchers need to ensure proper posture, handling of the instruments, and calibrating instruments. On the contrary, if the researchers have a more straightforward measurement task and formula through which handgrip power can be predicted near perfect for a population, that will ease the process. Formulating the equation requires considering all the relevant factors influencing the handgrip power and incorporating those factors in the formula to predict.

Factors influencing handgrip strength have been a topic of interest to researchers for the practical application of grip strength. Researchers found that an individual’s handgrip power was influenced by height, weight, dominant hand, forearm girth, hand length, and handbreadth [23–26]. Several researchers described the forearm and hand measurements as better predictors of maximum grip strength than height and weight [27, 28]. Age, gender, ethnicity, occupation, social status, lifestyle, and psychosocial variables influence grip power [29–36]. Ethnic variation in the population has been reported to influence anthropometry. Dimensions of upper limb bones vary in different ethnicity, gender, and age groups and with the opposite side of the body [37]. Hence, each population should have its model to predict handgrip power.

In northern Borneo, Sabah, the East Malaysian state, is renowned for its rich cultural and environmental diversity. Sabah has over fifty main ethnic groups with their languages [38]. KadazanDusun, Bajau, and Malay (Bruneian) ethnic groups are the majority among the ethnic groups in the Sabahan population. At the same time, the Chinese made up the largest non-indigenous group in Sabah [39]. Very few anthropometric works have been done among the major ethnic groups of Sabah. A study on the Malay, Indian and Chinese ethnicity of the Peninsular Malaysian population revealed that the dominant handgrip strength was positively associated with height and body mass index and negatively associated with age for both sexes. Dominant handgrip strength was related to work status for men but not for women. However, there was no difference in grip strength among ethnic groups [29].

With the above perspective, the present study was carried out to investigate the presence of any linear relationship of handgrip power with hand anthropometric variables (hand length, handbreadth, middle finger length, second inter-crease length of the middle finger, and hand span), gender, and ethnicity in young adults of Sabah.

## Methods

This cross-sectional study was carried out from January 2020 to December 2021 in the Anatomy Unit of the Faculty of Medicine and Health Sciences, Universiti Malaysia Sabah.

### Sample selection

Upon selecting the participants for the study for obtaining more valid information, specific inclusion criteria were imposed in selecting the participants. The participants qualified for being included in the research by fulfilling the following criteria:

- The age range must be 18–25 years old
- They are from KadazanDusun, Bajau, Malay (Bruneian), and Chinese ethnicity.
- They have a normal BMI.
- They reside within the university campus.
- They lead sedentary lifestyles.

The exclusion criteria were as follows:

- Individuals who had medical conditions (for example, stroke, rheumatoid arthritis, parkinsonism, and any other conditions that may affect the result) affect hand anthropometry and handgrip power.
- Known players of sports that require gripping and engaging handgrip strength.

### Study population

The participants were the students and staff of the University who hail from Kudat, Kota Belud, Tuaran, Ranau, Tamparuli, and Papar, as the desired ethnic groups for this study resided there. For example, mostly Bajau can be found at Kudat, Tuaran, Kota Belud, and Papar. While mostly KadazanDusun and Malay can be found at Tuaran, Ranau, Papar, and Tamparuli, and Chinese can be found in Kudat [39]. The participants’ parents and grandparents were required to be from the same ethnic group.

### Sample size

The minimum sample size recommended by the researcher is 25 per stratum [40]. The researchers anticipated a 50% response rate from previous experience in obtaining subjects from the same population and invited 50 persons per stratum. Later on, 46 persons per stratum participated in the study, which amounted to [46 × 4 (KadazanDusun, Bajau, Malay, and Chinese) × 2 (male and female)=] 368 persons.

### Sampling of the subject

This study applied a stratified random sampling method. There were eight strata in the sampling: ethnicity (KadazanDusun, Bajau, Malay, and Chinese) and gender (male and female). At first, a list of names of the adult Sabahan population (18–25 years) who belonged to the desired ethnicity was obtained from Bahagian Perkhidmatan Akademik (BPA). Then, the population was stratified into four ethnicities and was further stratified as males and females. Then, 46 subjects were randomly selected from a list of numbers picked randomly from a container until each gender and ethnic group met the intended sample size.

### Data collection

The study design, objective, and methodology were explained to the respondent, and informed consent was obtained from them. The hand dimensions were measured using INSIZE (0–200 mm × 0.01 mm 1108–200) digital calliper. The value was recorded in centimetres to the nearest 0.1 cm. The measurement was repeated two times, and an average was taken. The handgrip power was measured using a CAMRY (model no.: EH101) portable dynamometer. Researchers stated that the digital Camry dynamometer could be interchanged with the hydraulic Jamar hand dynamometer in the 40–59-year-old sub-group [41].

### Measurement

#### Hand length [42]

The hand’s length was measured as the straight distance from the midpoint of the distal wrist crease to the most distal point of the middle finger.

#### Handbreadth [42]

The hand’s breadth was measured as the hand’s width from the lateral surface of metacarpal II to the medial surface of metacarpal V. The hand’s breadth was measured at the level of the knuckles.

#### Middle (third) finger length [43]

Measurement of the middle finger was taken from the proximal finger crease of the middle (third) finger to the tip of the middle (third) finger.

#### Second inter crease length of the middle (third) finger [44]

Second inter crease length (middle phalanx) was measured from the distal interphalangeal joint crease to the proximal interphalangeal joint crease.

#### Measurement of the hand span [45]

Handspan was measured from the tip of the thumb to the tip of the small finger, with the hand spreading as wide as possible.

#### Handgrip power

During each handgrip strength measurement, the subjects were ensured to stand on both legs relaxed and put equal weight on both feet. Their feet were placed apart at shoulder width breadth, and shoulders were in vertical adduction with neutral rotation; elbows were flexed at 90° and forearms in a neutral position, wrists between 0°–30° dorsiflexion and 0°–15° ulnar deviation. The dynamometer was adjusted in the third position of the handle [41]. Participants were verbally motivated to continue using their maximum strength [46]. The dynamometer measures the highest value reached within three seconds. At first, the right hand (RHGP) and then left hand (LHGP) grip strength were evaluated using three repetitions [47]. Each repetition was evaluated with 1-min rests between measurements. There was a 5-min rest before evaluating the left-hand grip power. The attempt with the highest measurement out of the ten repetitions was recorded in kilogram as maximum strength [48].

#### Dominant hand

In the study, the dominant hand of the subjects was determined based on the difference in handgrip strength. Those who demonstrated significantly higher handgrip strength for the right hand were considered right-handed, and those who demonstrated either more for the left hand or no significant difference between the hands were considered left-handed [49].

### Statistical analysis

An unpaired *t* test was used to investigate the differences in the mean between the gender, and one-way ANOVA was used to investigate the same between ethnicities. The relationship between the response variable and explanatory variables was analysed at first through simple linear regression and then multiple linear regression. Multicollinearity between the numeric variables was examined by the Pearson’s correlation test. *R*^2^, adjusted *R*^2^, and standard errors of the estimates were used to compare different models. Statistical analyses were performed using IBM SPSS Statistics 27 and StatCrunch. The level of significance α = 0.05 was chosen to avoid type II error in attempting to choose a very small α.

## Results

Among the 368 participants, more than two-thirds were right-handed, and this distribution was observed among all the subgroups except the Malay males, where this ratio was comparatively less (Table 1).

**Table 1:** Frequency distribution of hand dominance among the respondents (*n* = 368)

Ethnicity	Gender	Left-handed ***n*** (%)	Right-handed ***n*** (%)	Total ***n*** (%)
**KadazanDusun**	**Male**	8 (17.39%)	38 (82.61%)	46 (100%)
	**Female**	8 (17.39%)	38 (82.61%)	46 (100%)
**Bajau**	**Male**	11 (23.91%)	35 (76.09%)	46 (100%)
	**Female**	10 (21.74%)	36 (78.26%)	46 (100%)
**Malay**	**Male**	17 (36.96%)	29 (63.04%)	46 (100%)
	**Female**	14 (30.43%)	32 (69.57%)	46 (100%)
**Chinese**	**Male**	9 (19.57%)	37 (80.43%)	46 (100%)
	**Female**	9 (19.57%)	37 (80.43%)	46 (100%)
**Total**		86 (23.37%)	282 (76.63%)	368 (100%)

A right-handed female and a male, on average, RHGP of 25.41 kg and 41.32 kg, while a left-handed female and a male had 23.98 and 37.72 kg of LHGP, respectively (Table 2).

**Table 2:** Distribution of handgrip power according to dominant hand among the participants (*n* = 368)

Dominant hand	Gender	Handgrip power	Mean ± SD (kg)	95% CI	
				Lower bound	Upper bound
**Right**	**Male** (*n* = 139)	RHGP	41.32 ± 7.34	40.09	42.56
		LHGP	36.54 ± 7.04	35.35	37.72
	**Female** (*n* = 143)	RHGP	25.41 ± 5.24	24.54	26.27
		LHGP	21.91 ± 4.74	21.13	22.70
**Left**	**Male** (*n* = 45)	RHGP	34.89 ± 7.35	32.68	37.10
		LHGP	37.72 ± 7.14	35.57	39.86
	**Female** (*n* = 41)	RHGP	22.42 ± 4.76	20.92	23.92
		LHGP	23.98 ± 5.09	22.38	25.59

*LHGP* left handgrip power, *RHGP* right-hand grip power

Among the males, Bajau males had the highest mean RHGP (41.83 ± 7.28) and LHGP (39.17 ± 7.39), whereas among the females, KadazanDusun females had the highest mean RHGP (25.82 ± 5.97) and Chinese females had the highest mean LHGP (22.93 ± 4.78). Malay males’ mean RHGP (38.58 ± 8.06) was the lowest among males, while Chinese males’ mean LHGP (35.01 ± 6.78) was the lowest. The lowest means of RHGP and LHGP belonged to Malay females (23.85 ± 4.50) and Bajau females (21.75 ± 4.98), respectively (Table 3).

**Table 3:** Distribution of central tendency and confidence interval of handgrip power according to the gender and ethnicity of the respondents (*n* = 368)

Ethnicity	Gender	Handgrip power	Mean ± SD (kg)	95% CI	
				Lower bound	Upper bound
**KadazanDusun**	Male (*n* = 46)	RHGP	39.77 ± 7.67	37.49	42.05
		LHGP	36.74 ± 6.67	34.76	38.73
	Female (*n* = 46)	RHGP	25.82 ± 5.97	24.05	27.59
		LHGP	22.83 ± 5.16	21.29	24.36
**Bajau**	Male (*n* = 46)	RHGP	41.83 ± 7.28	39.67	43.99
		LHGP	39.17 ± 7.39	36.97	41.36
	Female (*n* = 46)	RHGP	24.03 ± 5.85	22.29	25.76
		LHGP	21.75 ± 4.98	20.28	23.23
**Malay**	Male (*n* = 46)	RHGP	38.58 ± 8.06	36.19	40.97
		LHGP	36.38 ± 6.97	34.31	38.45
	Female (*n* = 46)	RHGP	23.85 ± 4.50	22.51	25.18
		LHGP	21.99 ± 4.66	20.60	23.37
**Chinese**	Male (*n* = 46)	RHGP	38.70 ± 7.82	36.38	41.03
		LHGP	35.01 ± 6.78	32.99	37.02
	Female (*n* = 46)	RHGP	25.28 ± 4.48	23.95	26.61
		LHGP	22.93 ± 4.78	21.51	24.36

*LHGP* left handgrip power, *RHGP* right-hand grip power

In Table 3, the mean value of handgrip power for male participants was more than for females for both hands. An independent sample *t* test was performed to investigate this observation. The hypothesis was determined to start the investigation,

- H_0_: There is no difference between male and female handgrip power among the participants.
- H_1_: Male participants have higher handgrip power than female participants.

As the participants were recruited using stratified random sampling, the observations were independent; the number of male and female participants was more than 30 persons and was no more than 5% of the population, so the sample fulfilled the assumptions for the intended *t* test [50].

The *p* value for the *t* statistics for the difference of means for handgrip power of both sides is < 0.001 (Table 4), which is less than the level of significance, α = 0.05. So, the null hypothesis is rejected. Thus, the sample suggests sufficient evidence to conclude that male participants had higher handgrip power than females.

**Table 4:** Difference in handgrip power among the participants concerning gender (*n* = 368)

	Gender	Mean (±SD)	t	DF	***P*** value	Mean difference	Std. error difference	95% CI of the difference	
								Lower	Upper
**RHGP**	**Male** (*n* = 184)	39.72 ± 7.76	21.661	366	< .001	14.98	.691	13.62	16.34
	**Female** (*n* = 184)	24.74 ± 5.27							
**LHGP**	**Male** (*n* = 184)	36.82 ± 7.06	22.822	366	< .001	14.44	.633	13.20	15.69
	**Female** (*n* = 184)	22.38 ± 4.88							

*LHGP* left handgrip power, *RHGP* right-hand grip power

While observing the mean values of handgrip power among the ethnicities, some apparent differences were observed. The one-way AONVA test was conducted to test the following hypothesis:

- H_0_: There is no difference in handgrip power among the participants from Bajau, KadazanDusun, Malay and Chinese ethnicities.
- H_1_: Participants from at least one ethnicity have different handgrip powers than others.

**Fig. 1:**
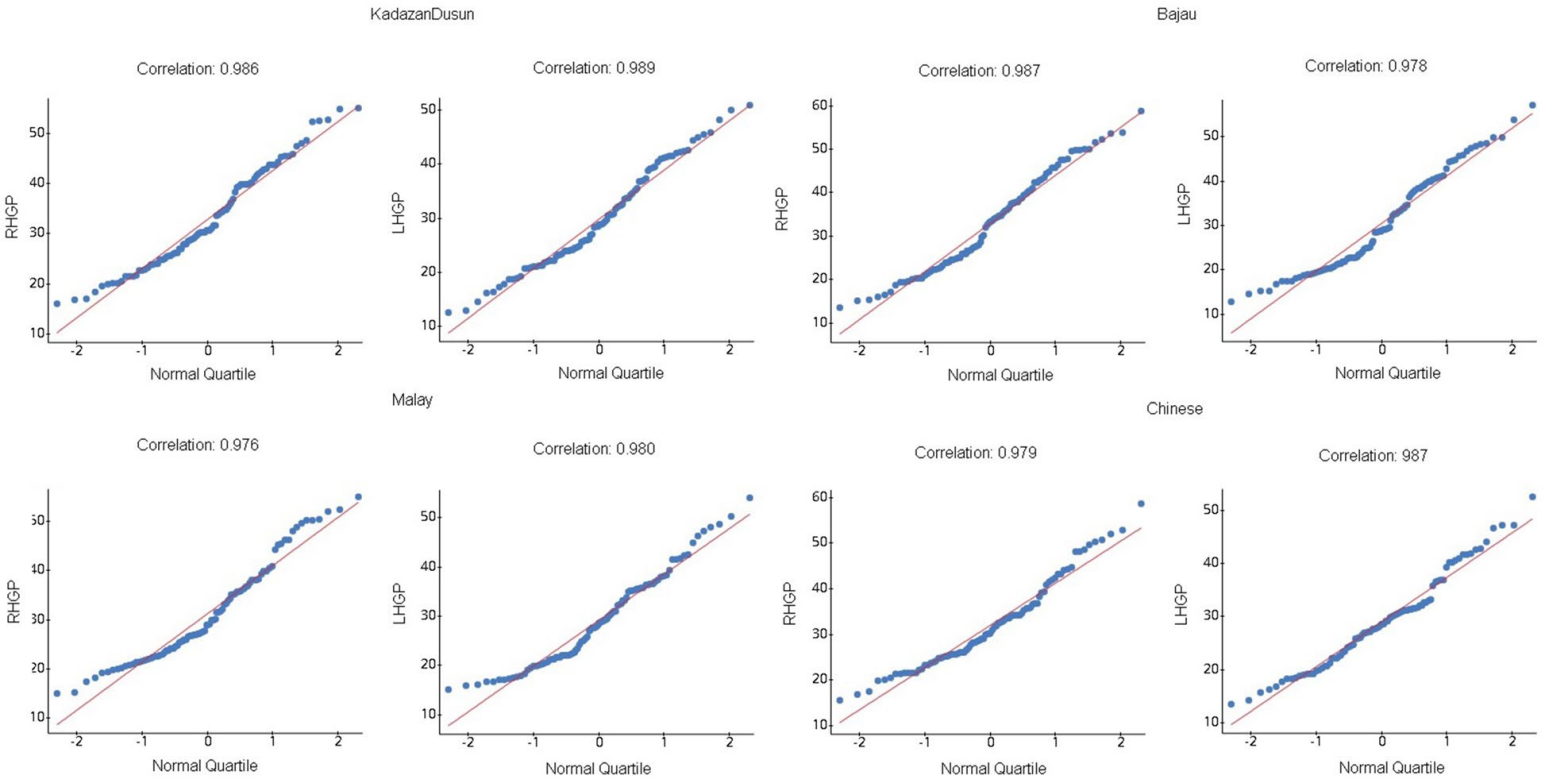
Q-Q plot showing the correlation between observations in different ethnicities with z-score

Other than the samples being randomly selected and independent, the one-way ANOVA test requires that the populations from where the samples were obtained are normally distributed, and the populations must have the same variance [50]. The normal probability plots for each ethnicity were drawn in StatCrunch, along with the correlation between the score and expected *z*-score, demonstrated in Fig. 1.

The correlation values are higher than 0.960, the critical value for a sample size of more than 30 [51]. Therefore, it is reasonable to conclude that each data set comes from a normally distributed population. Again, for the assumption of having an equal variance, the standard deviations were compared where the largest standard deviation, 8.06 smaller than twice the smallest, 4.48 (4.48 × 2 = 8.96 > 8.06), the requirement of equal population variances is satisfied.

**Fig. 2:**
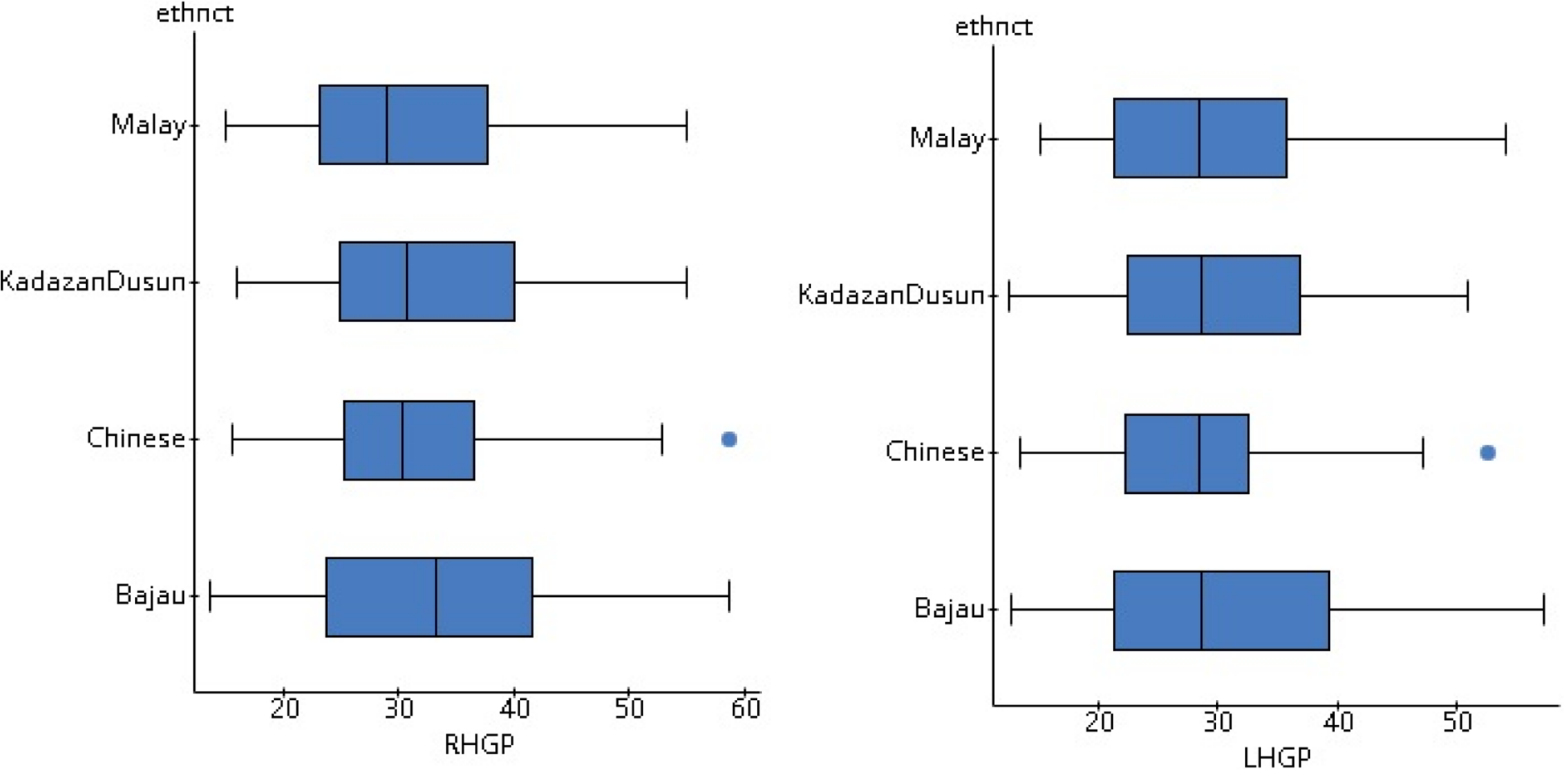
Boxplot showing the comparison of handgrip powers between participants from different ethnicities

The results from Table 5 show that *p* values for right and left-hand grip powers are 0.629 and 0.729, respectively. As these *p* values are more than the level of significance α = 0.05, the null hypothesis is retained. There is insufficient evidence to conclude that there is a difference in handgrip power among the participants from Bajau, KadazanDusun, Malay, and Chinese ethnicities. The box plot in Fig. 2 supports the ANOVA result.

**Table 5:** Difference in handgrip power among the participants concerning ethnicities (*n* = 368)

		Sum of squares	DF.	Mean square	***F***	***P*** value
**RHGP**	**Between ethnicities**	174.361	3	58.120	.579	.629
**LHGP**	**Between ethnicities**	123.534	3	41.178	.460	.710

*LHGP* left handgrip power, *RHGP* right-hand grip power

In Fig. 2, for Chinese ethnic group participants’ data, two outliers might confuse for violating the assumption of normality of data. Q-Q plots were drawn with the corresponding residuals using StatCrunch to verify the normality of the data. The plots were approximately linear, and correlation statistics for right-hand (0.979) and left-hand (0.987) were more than 0.960, the critical value for sample size more than 30 [51]. It is reasonable to conclude that the residuals are normally distributed. So, despite having outliers, the data were normally distributed.

A correlation test was performed to investigate any association between the handgrip power and the explanatory variables considered in this study. Among the explanatory variables explanatory, gender and dominant hands are categorical variables. Spearman rank correlation test was performed to test the association as it does not require the assumption that the data is continuous and normally distributed as Pearson correlation requires [52]. From the correlation matrix in Table 6, it can be commented that other than ethnicity and left-handedness, all other explanatory variables had a statistically significant correlation with the handgrip power. Even though there was a significant correlation, the correlation coefficients for the right-handedness, right, and left second inter-crease lengths were weak [52].

**Table 6:** Relationship between handgrip power, height, and hand anthropometry of the respondents (*n* = 368)

	RHGP		LHGP	
	***r***	***p*** value	***r***	***p*** value
**Height**	0.565	< .001	0.564	< .001
**RHL**	0.631	< .001		
**RHB**	0.744	< .001		
**RHS**	0.489	< .001		
**RMFL**	0.557	< .001		
**R2ICL**	0.337	< .001		
**LHL**			0.636	< .001
**LHB**			0.731	< .001
**LHS**			0.503	< .001
**LMFL**			0.563	< .001
**L2ICL**			0.363	< .001

*LHL* left-hand length, *LHB* left handbreadth, *LHGP* left handgrip power, *LMFL* left middle finger length, *L2ICL* left second inter-crease length, *LHS* left-hand span, *RHL* right-hand length, *RHB* right handbreadth, *RHS* right-hand span, *RHGP* right-hand grip power, *RMFL* right middle finger length, *R2ICL* right second inter-crease length

**Fig. 3:**
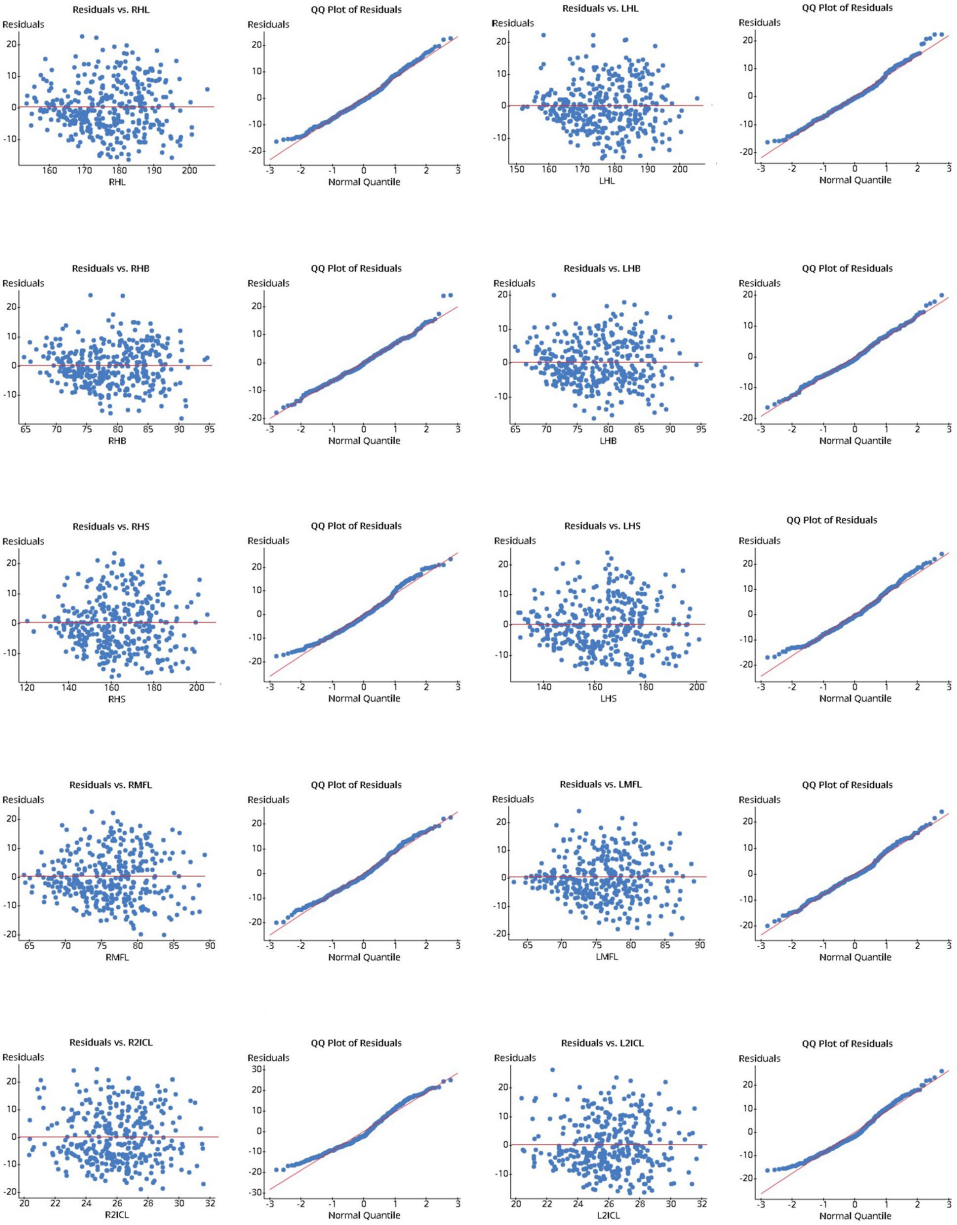
Scatter plots and Q-Q plots residuals (simple linear regression) against different hand dimensions

The relationship between hand length, breadth, span, middle finger length, second inter-crease length, and handgrip power was examined first through a simple linear regression model and finally with a multiple linear regression model. As the participants were randomly selected, the first assumption of linear regression was achieved. The linear regression model assumes a relationship between the explanatory variable (hand dimensions individual) and the response variable (handgrip power). The distribution of the residuals against each parameter was constructed to observe the normal probability plot for linear relation, and correlation statistics were also calculated (Fig. 3). All the residuals demonstrated an approximately linear relationship with z-score, and correlation statistics for the residuals against all hand dimensions were higher than 0.960, the critical value for sample size more than 30 [51]. So, it is reasonable to conclude that the hand power was normally distributed for each hand dimension.

The residuals were plotted against the hand dimensions to verify the last requirement of constant error variance. In the residual plot in Fig. 3, the residuals are evenly spread around a horizontal line drawn at zero. As the model has constant error variance, statistical inference using the regression model is reliable. So, the requirement of constant variance is satisfied [50].

As the data were appropriate for linear regression, the following hypotheses were created to test the relationship between hand length, breadth, span, middle finger length, and second inter-crease length of middle finger with handgrip power.

- H_0_: There is no linear relation (β_i_ = 0), H_1_: There is a linear relationship (β_i_ ≠ 0)

The intercept (β_0_), slope (β_1_), the *p*-value for the slope, correlation coefficient, coefficient of determination (*R*^2^), adjusted coefficient of determination (Adj.*R*^2^), standard error of estimate (SEE), and ANOVA test for the model with *p* value was determined using StatCrunch. As the *p* values (< 0.001) for the slope were less than the level of significance α = 0.05 (Table 7), the null hypothesis is rejected. There is sufficient evidence at the α = 0.05 level of significance to conclude that a linear relation exists between hand length, breadth, span, middle finger length, second inter-crease middle finger length, and hand power.

**Table 7:** Intercepts, coefficients, and relationships of hand dimensions with handgrip power (*n* = 368)

Response variable	Explanatory variable	Intercept (β_**0**_)	Slope (β_**i**_)	Correlation Coefficient (r)	***P*** value
**RHGP**	**Height**	− 77.410	0.679	0.569	< 0.0001
	**RHL**	− 73.747	0.599	0.631	< 0.0001
	**RHB**	− 67.762	1.268	0.744	< 0.0001
	**RHS**	− 18.641	0.311	0.487	< 0.0001
	**RMFL**	− 53.704	1.129	0.557	< 0.0001
	**R2ICL**	− 6.939	1.507	0.335	< 0.0001
**LHGP**	**Height**	− 73.429	0.638	0.567	< 0.0001
	**LHL**	− 71.347	0.571	0.636	< 0.0001
	**LHB**	− 64.553	1.202	0.731	< 0.0001
	**LHS**	− 21.122	0.310	0.503	< 0.0001
	**LMFL**	− 50.576	1.053	0.563	< 0.0001
	**L2ICL**	− 11.611	1.579	0.370	< 0.0001

*LHL* left-hand length, *LHB* left handbreadth, *LHGP* left handgrip power, *LMFL* Left Middle finger length, *L2ICL* left second inter-crease length, *LHS* left-hand span, *RHL* right-hand length, *RHB* right handbreadth, *RHS* right-hand span, *RHGP* right-hand grip power, *RMFL* right middle finger length, *R2ICL* right second inter-crease length

Simple linear models for each hand dimension were constructed using the intercept and slope (Table 7). The right-hand breadth (RHB) had the highest coefficient of correlation (*r* = 0.744) and coefficient of determination (*R*^2^ = 0.554), which indicates that 55.4% of the variation of right-hand grip power (RHGP) is explainable by the variation of RHB with the lowest SEE (6.69) indicating that RHB is the most reliable for estimating RHGP.

The left-hand breadth (LHB), on the other side, had a coefficient of correlation (*r* = 0.731) and the highest coefficient of determination (*R*^2^ = 0.535), which indicates 53.5% of the variation of left-hand grip power (LHGP) is explainable by the variation of LHB with SEE of 6.45 which the lowest among left-hand parameters and an indicator for being the most reliable for estimating LHGP.

Table 8 demonstrates that hand length, handbreadth, hand span, middle finger length, and second inter-crease length demonstrated a linear relationship with the handgrip power of the respective side.

**Table 8:** The goodness-of-fit measure of simple linear models predicts hand grip power from hand dimensions

Explanatory variables	Model	***R***^**2**^	Adj. ***R***^**2**^	SEE.	***F***	***P*** value
**Height**	− 77.410 + 0.679 Height	0.324	0.322	8.24	175.21	< 0.0001
**RHL**	− 73.052 + 0.747 RHL	0.399	0.396	7.68	242.78	< 0.0001
**RHB**	− 67.762 + 1.268 RHB	0.554	0.552	6.69	454.33	< 0.0001
**RHS**	− 18.641 + 0.311 RHS	0.239	0.237	8.74	114.77	< 0.0001
**RMFL**	− 53.704 + 1.129 RMFL	0.310	0.309	8.32	164.66	< 0.0001
**R2ICL**	− 6.939 + 1.507 R2ICL	0.112	0.111	9.44	46.16	< 0.0001
**Height**	− 73.429 + 0.638 Height	0.321	0.318	7.78	173.15	< 0.0001
**LHL**	− 71.347 + 0.571 LHL	0.404	0.403	7.30	248.38	< 0.0001
**LHB**	− 64.553 + 0.731 LHB	0.535	0.534	6.45	420.91	< 0.0001
**LHS**	− 21.122 + 0.310 LHS	0.253	0.251	8.17	124.08	< 0.0001
**LMFL**	− 50.576 + 1.053 LMFL	0.317	0.315	7.81	170.10	< 0.0001
**L2ICL**	− 11.611 + 1.579L2ICL	0.137	0.129	8.78	58.17	< 0.0001

*Adj. R*^*2*^ adjusted R-squared, *LHL* left-hand length, *LHB* left handbreadth, *LMFL* left middle finger length, *L2ICL* left second inter-crease length, *LHS* left-hand span, *RHL* right-hand length, *RHB* right handbreadth, *RHS* right-hand span, *RMFL* right middle finger length, *R2ICL* right second inter-crease length, *SEE* standard error of estimate

As there was no significant correlation between handgrip power and the ethnicities but in-between gender was, gender was included in the linear model as an indicator variable where a male was coded as 0 and a female as 1.

Multiple linear regression is required to test the relationship between height, hand dimensions, and gender with handgrip power. The residuals of the test are required to be normally distributed and the absence of an outlier to draw inference on the findings of multiple regression. The test also requires avoiding multicollinearity [51]. The correlation matrix between the explanatory variables was performed, and the results are tabulated in Table 9.

**Table 9:** Correlation matrix

	Gender	RHL	RHB	RHS	RMFL	R2ICL		Gender	LHL	LHB	LHS	LMFL	L2ICL
**RHL**	− 0.683						**LHL**	− 0.695					
**RHB**	− 0.759	0.769					**LHB**	− 0.752	0.780				
**RHS**	− 0.567	0.610	0.627				**LHS**	− 0.545	0.619	0.616			
**RMFL**	− 0.558	0.901	0.678	0.556			**LMFL**	− 0.592	0.907	0.693	0.580		
**R2ICL**	− 0.324	0.677	0.432	0.371	0.768		**L2ICL**	− 0.371	0.710	0.478	0.377	0.579	
**Height**	− 0.729	0.776	0.606	0.523	0.693	0.519	**Height**	− 0.729	0.782	0.616	0.490	0.704	0.540

*LHL* left-hand length, *LHB* left handbreadth, *LHGP* left handgrip power, *LMFL* left middle finger length, *L2ICL* left second inter-crease length, *LHS* left-hand span, *RHL* right-hand length, *RHB* right handbreadth, *RHS* right-hand span, *RHGP* right-hand grip power, *RMFL* right middle finger length, *R2ICL* right second inter-crease length

The hand lengths had a high correlation with middle finger lengths and handbreadths. As handbreadth demonstrated the highest relationship with handgrip power, hand lengths, and middle finger lengths were removed from the regression test to avoid the effect of multicollinearity,

The hypothesis to be tested by multiple regression is

- H_0_: There is no relation between handbreadth, hand span, second inter-crease length, gender, and handgrip power (β_1_ = β_2_ = β_3_ = β_4_ = 0)
- H_1_: There is a linear relation between hand dimensions, gender, and handgrip power (at least one β_i_ ≠ 0)

**Fig. 4:**
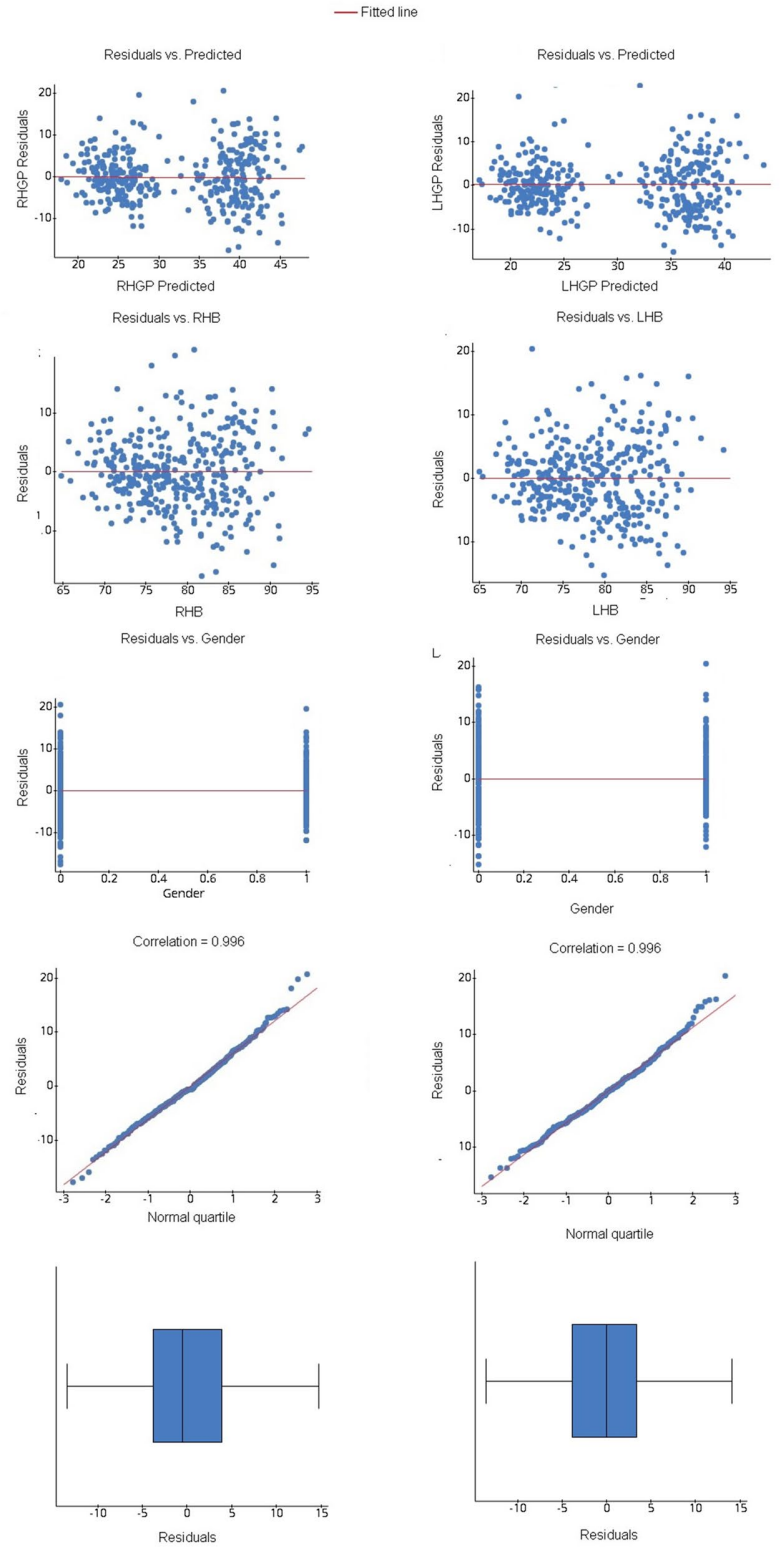
Residual plots for assumptions of multiple linear regression

As the *p* values of the slopes are much below the significant level (Table 10), the null hypothesis is rejected. Before drawing an inference of the finding, the assumptions of the normality of the residuals, equal distribution of the variances, and absence of outliers were ensured (Fig. 4).

**Table 10:** Intercepts and estimates of the multiple linear regression model

Response variable	Parameter	Estimate	Std. err.	DF	***T*** Stat	***P*** value
RHGP	Intercept	− 18.972	6.921	365	− 2.741	0.0064
	Gender	− 8.574	0.975	365	− 8.952	< 0.0001
	RHB	6.398	0.938	365	8.499	< 0.0001
LHGP	Intercept	− 11.621	6.467	365	− 1.797	0.0732
	Gender	− 9.389	0.896	365	− 10.481	< 0.0001
	LHB	5.861	0.781	365	7.507	< 0.0001

*LHGP* left handgrip power, *RHGP* right-hand grip power

**Fig. 5:**
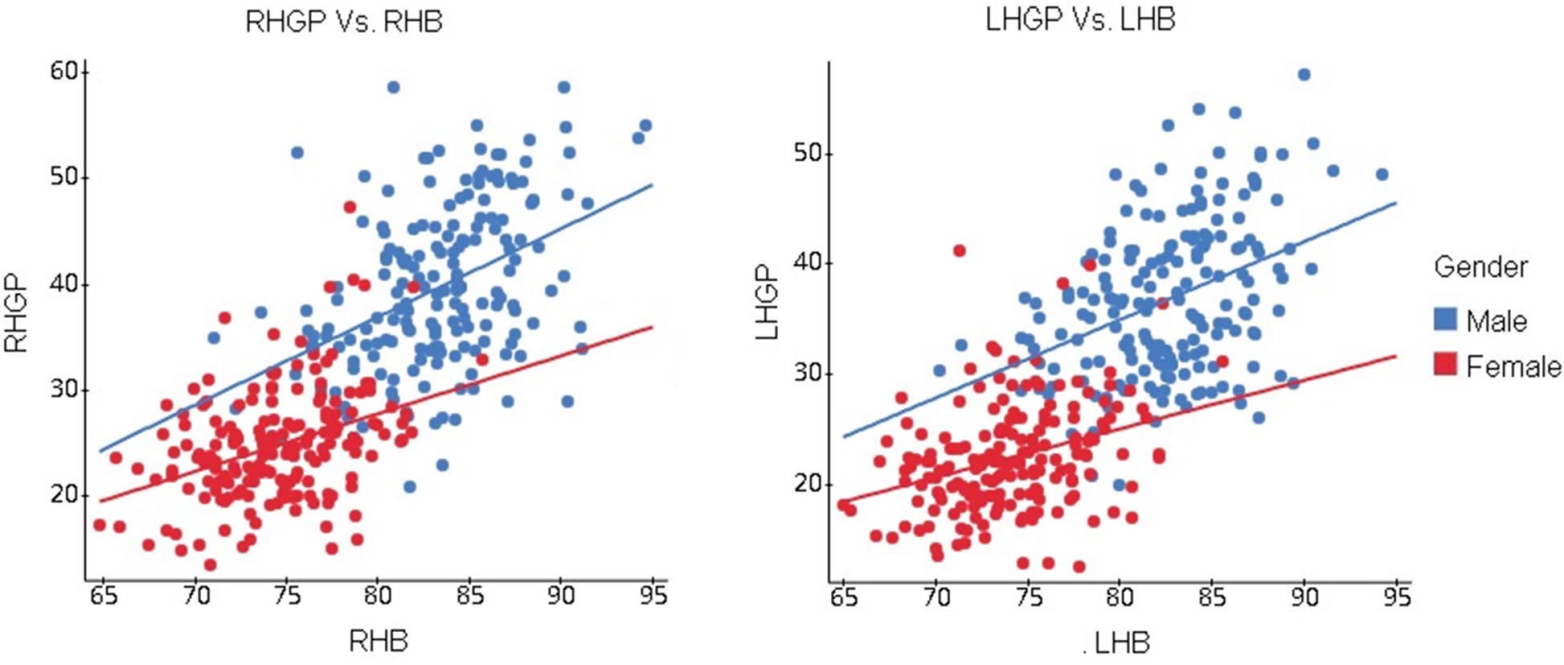
Scatter diagram showing the relationship of RHB and LHB with RHGB and LHGB in males and females

Hence, sufficient evidence concludes that RHB and gender have a linear relation with RHGP while LHB and gender have a linear relation with LHGB (Fig. 5).

The values for the *F*-statistics are below the significance level (*p* < 0.05), and from Fig. 4 normal distribution of the residuals indicate the appropriateness of the models (Table 11). If we put the value of the gender codes (male = 0, female = 1), we get the following formulas:

$${\displaystyle \begin{array}{l}\mathrm{Male}:\mathrm{RHGP}=-18.972+7.043\ \mathrm{RHB},\mathrm{LHGP}=-11.621+5.861\ \mathrm{LHB}\\ {}\mathrm{Female}:\mathrm{RHGP}=-27.676+7.043\ \mathrm{RHB},\mathrm{LHGP}=-21.01+5.861\ \mathrm{LHB}\\ {}\begin{array}{l}\mathrm{Right}-\mathrm{handed}\ \mathrm{male}:\mathrm{RHGP}=-17.566+0.089\mathrm{RHB},\mathrm{LHGP}=-15.773+0.084\mathrm{LHB}\\ {}\mathrm{Right}-\mathrm{handed}\ \mathrm{female}:\mathrm{RHGP}=-18.02+0.089\ \mathrm{RHB},\mathrm{LHGP}=-15.301+0.084\mathrm{LHB}\\ {}\begin{array}{c}\mathrm{Left}-\mathrm{handed}\ \mathrm{male}:\mathrm{RHGP}=-5.177+0.217\mathrm{RMFL},\mathrm{LHGP}=-0.496+0.244\mathrm{LHB}\\ {}\mathrm{Left}-\mathrm{handed}\ \mathrm{female}:\mathrm{RHGP}=-5.768+0.217\mathrm{RMFL},\mathrm{LHGP}=-1.06+0.244\mathrm{LHB}\end{array}\end{array}\end{array}}$$

**Table 11:** The goodness-of-fit measure of multiple linear regression models predicts hand grip power generally and based on the dominant hand

	Model	***R***^**2**^	Adj. ***R***^**2**^	***F***	***P*** value
	RHGP = − 18.972 − 8.704 Gender + 7.043 RHB	0.6342	0.6322	212.282	< 0.0001
	LHGP = − 11.621 − 9.389 Gender + 5.861 LHB	0.6425	0.6405	327.980	< 0.0001
Right-handed	RHGP = − 17.240 − 9.265 Gender + 6.979RHB	0.6832	0.6809	300.803	< 0.0001
	LHGP = − 15.773 − 8.907 Gender + 6.303LHB	0.6671	0.6648	279.605	< 0.0001
Left-handed	RHGP = 4.754 − 10.386 Gender + 3.829RMFL	0.5360	0.5248	47.940	< 0.0001
	LHGP = − 0.496 − 10.425 Gender + 4.558LHB	0.5796	0.5695	57.221	< 0.0001

*Adj. R*^*2*^ adjusted R-squared, *LHB* left handbreadth, *LHGP* left handgrip power, *RHB* right handbreadth, *RHGP* right-hand grip power

## Discussion

The current study adopted multiple linear regression to form a practical and achievable model and attempted to adjust the confounding factors. Researchers advised randomization, restriction, and matching at the study design level together with stratification and multivariate analysis at the statistical level to eliminate or adjust confounding [53]. The present study incorporated all possible ways to control the confounding. At first, the subjects were selected through stratified random sampling from four different ethnic groups and an equal number of males and females from each ethnic group. Then the subjects were restricted to a fixed age group who were students residing within the same campus, leading a sedentary lifestyle, and having a normal BMI. As it was impractical to match every stratum, multivariate analysis (multiple linear regression) models were adopted for adjusting the confounding at the statistical analysis level.

Some researchers demonstrated a relationship between the handgrip power and age groups in the Malaysian population [26, 29]. In a study, the subjects were grouped at 18–24 years, 25–34 years, 35–44 years, 45–54 years, and 55–65 years to find the relationship. The study demonstrated that the grip strength had a linear association with age, peaked in the 18 to 34 age group, and gradually decreased. The study could not establish a linear relationship in the dominant left-hand group owing to the smaller sample size but demonstrated maximum handgrip power in age-groups 18–24 years and 35–44 years [26]. Another study further demonstrated that handgrip power was progressively lower with increasing age after the fifth decade in both men and women. Nevertheless, there was no difference in handgrip strength among the groups 20–29 years, 30–39 years, and 40–49 years [33]. Another study even found the change after 70 years [34]. Hence, the present study included subjects 18 to 25 years of age, considering a group of subjects with similar handgrip strength.

Likewise, age, BMI also has been adjusted in this study. Among the studies conducted on the Malaysian population, one group of researchers could not find significant correlations between grip strength and BMI but found a significant correlation with weight and height individually using the Pearson test [26]. The other study, on the contrary, yielded a regression coefficient that demonstrated that height and BMI were positively related to grip strength for both sexes [29]. Another study on the Greek population did not observe any association between BMI and dominant handgrip power in the total study sample and male participants. However, the study found a moderate negative correlation between the dominant hand grip power and BMI in females [54].

The occupation of a person is a vital influencing factor for handgrip power. A study on the Korean population demonstrated that the mean strengths of both hands in subjects with more physically demanding occupations were greater than those of subjects with occupations with medium and low physical demands [55]. Hence, subjects of the current study were chosen from the students who were not involved in the games or activities that require physical strength, more precisely, gripping and engaging handgrip strength. Occupation is also an essential element of household income and social status. A study on African American and White adults revealed that for men aged ≤ 49 years, men in low-income households had lower grip strength than men in high-income households [36]. Subjects of this research are the students residing within the university campus where they lead similar income environments as those who lack are being supported by the university either in the form of scholarships or various support schemes.

The present study inspected the difference in handgrip power between the male and female respondents, where males demonstrated significantly higher handgrip powers than females (Table 3), which corresponds to various research findings [26, 29–31]. The present study also investigated different ethnicities’ differences in handgrip power (Table 4). While investigating the difference among the major ethnicities of Sabah, the present study demonstrated no significant difference in handgrip power among the KadazanDusun, Bajau, Malay, and Chinese populations, which corresponds to other studies on the Malaysian population [26, 29]. However, the handgrip power of the respondents of this study was comparatively higher than a previous study done on the Malay, Chinese, and Indian populations in West Malaysia [26]. Although the current study also had Malay and Chinese populations within the sample frame, their origin is not like West Malaysia. In East Malaysia, the Malays are mostly of Bruneian and Kadayan origin [38] while Malay sub-ethnic groups in peninsular Malaysia are Melayu Kelantan, Melayu Minang, Melayu Jawa, and Melayu Bugis [56]. Among the Chinese population, the Hakkas are prominent in Sabah, along with Cantonese, Hokkien, Teochew, Hainanese, and Shantung [38], whereas, in West Malaysia, Hokkien, Cantonese, Foochows, and other groups are the majority [57]. This difference in their origin might have played a role in the difference in handgrip strength. So, there was no difference while comparing the ethnicities within the same geographical area, but the difference was significant while comparing the same ethnicities [26, 29] from different geographical areas. On the contrary, some researchers demonstrated ethnic variation influencing handgrip power within the same geographical area. In a study, African Americans exhibited stronger grip strength than Whites [35]. Another study demonstrated Non-Hispanic blacks and Hispanics had higher handgrip power when compared to Non-Hispanic whites [36].

In a study on the Malaysian population, the handgrip power was distributed based on the dominant hand [29]. The current study’s findings correspond to all right-handed males and right and left-handed females of that study. However, left-handed males in that study demonstrated higher handgrip strength than the same subjects in the present study. Since all the subjects’ handgrip power was demonstrated as average and was not stratified into different ethnicities, this type of generalization might have contributed to the similarity. On the contrary, the lower number of left-handed subjects in the current study than the same group of subjects in that study might be the reason for the disparity.

The study examined the relationship between handgrip power, gender, ethnicity, handedness, height, and hand dimensions (length. breadth, span, middle finger length, and second inter-crease length of middle finger) to assess the possibility of using regression equations to predict handgrip strength from the explanatory variables. Among the qualitative explanatory variables, gender (*r* = − 0.778) demonstrated the highest relationship with handgrip power. A similar finding was demonstrated in a study on the French population [58]. Another study on 20–25 year-old young German males and females demonstrated similar findings where gender significantly influenced handgrip power [59]. The apparent difference in handgrip power between males and females could have influenced the handgrip power.

The highest association was observed among quantitative explanatory variables between handbreadth and handgrip power (on the right side, *r* = 0.798 and the left side, *r* = 731). A study on Indian inter-university softball players aged 17 to 25 demonstrated a significant correlation between right handbreadth and length with grip strength [60]. Another study on Bangladeshi cricket team batters showed a significant correlation between the handbreadth and handgrip power for both hands [61]. On the contrary, the study on the 20 to 25-year-old German population did not significantly affect hand length and handbreadth on handgrip power [59].

The forward selection method was adopted for multiple regression in the current study, where a variable was selected to enter the model if *p* ≤ 0.05. The study proposed general population models when they were not stratified based on hand dominance and stratification. Under these criteria, gender and handbreadth entered the model for all subjects when not stratified according to hand dominance, right-handed subjects, and left-handed subjects (predicting LHGP). Only right middle finger length entered instead of the handbreadth in the model predicting RHGP for left-handed subjects, although statistically significant, described the handgrip power’s lowest (53%) variability. The highest percentage (68% and 67%) of handgrip variability was demonstrated by the model predicting handgrip power for right-handed subjects, followed by the general models, which explained 63% and 64% of the variability of handgrip power. The study performed in West Malaysia on the Malay, Chinese and Indian populations estimated models to predict handgrip for both genders based on height, weight, and BMI [29]. However, the study did not reveal the percentage of variability explained by the models. However, another group of researchers predicted handgrip strength from height and weight for both genders, where the r-squared value ranged from 0.11 to 0.29 [26]. In another study on Malaysian populations where age, height, job groups, and diabetes significantly predicted handgrip strength in the multivariate model for males, while age, weight, height, and diabetes were the significant predictors for females [62]. The model for males explained 35% of the variability of handgrip power, whereas the model for females explained around 18%. A study on the French population predicted handgrip from hand circumferences, where the study could explain 68% of the variability of maximal handgrip strength [58].

From the literature review, it is evident that hand circumference is a good predictor of handgrip power. Although the present study did not include hand circumference in the model, it could explain more than 60% variability in general and even more in dominant right-hand persons of the Sabahan population. The remaining unexplained variability might be due to some missing explanatory variables like hand circumference, different age groups, and others. Again, the study was meant to be conducted at the community level, but due to COVID-19, data collection at the community level was not possible. Instead, the study included the students of the University who fulfilled the inclusion criteria and were fully vaccinated, which may not be representative of the major ethnic groups of Sabah. Nonetheless, the subjects were from different parts and major ethnic groups of Sabah, and they had similar lifestyles, food habits, and cultures. Again, while selecting samples, the medical conditions that might influence hand anthropometry and handgrip power were screened using a questionnaire, and no investigation confirmed it.

Hence, right and left handgrip power of 18 to 25 years old males from major ethnic groups of Sabah can be predicted using the models RHGP = − 18.972 + 7.043 RHB, and LHGP = − 11.621 + 5.861 LHB and for females, RHGP = –27.676 + 7.043 RHB and LHGP = − 21.01 + 5.861 LHB respectively.

## Conclusions

The predicted handgrip power would be a key to selecting a better player or a better worker or assessing the prognosis of a disease or the wellbeing of a person. The study can be further expanded to all ethnicities and ages of people of Sabah or even Malaysia.

## Data Availability

The datasets used and analyzed during the current study are available from the corresponding author on reasonable request.

## Abbreviations

Adj.R^2^: Adjusted R-squared
LHL: Left-hand length
LHB: Left handbreadth
LHGP: Left handgrip power
LMFL: Left middle finger length
L2ICL: Left second inter-crease length
LHS: Left-hand span
RHL: Right-hand length
RHB: Right handbreadth
RHS: Right-hand span
RHGP: Right-hand grip power
RMFL: Right middle finger length
R2ICL: Right second inter-crease length
SEE: Standard error of estimate

## References

[1] Jordre B, Schweinle W (2020). Hand grip strength in senior athletes: normative data and community-dwelling comparisons. Int J Sports Phys Ther.

[2] Fallahi AA, Jadidian AA. The effect of hand dimensions, hand shape and some anthropometric characteristics on handgrip strength in male grip athletes and non-athletes. J Hum Kinet. 2011. 10.2478/v10078-011-0049-2. 10.2478/v10078-011-0049-2 PMC3588620 23486361

[3] Taha NZ (2005). Grip strength prediction for Malaysian industrial workers using artificial neural networks. Int J Ind Ergon.

[4] Hogrel JY. Grip strength measured by high precision dynamometry in healthy subjects from 5 to 80 years. BMC Musculoskelet Disord. 2015. 10.1186/s12891-015-0612-4. 10.1186/s12891-015-0612-4 PMC4460675 26055647

[5] Mgbemena NC, Aweto HA, Tella BA, Emeto TI, Malau-Aduli BS. Prediction of lung function using handgrip strength in healthy young adults. Physiol Rep. 2019. 10.14814/phy2.13960. 10.14814/phy2.13960 PMC6328910 30632320

[6] Rantanen T, Guralnik JM, Sakari-Rantala R (1999). Disability, physical activity, and muscle strength in older women: the women’s health and aging study. Arch Phys Med Rehabil.

[7] Alfaro-Acha A, Al Snih S, Raji MA (2006). Handgrip strength and cognitive decline in older Mexican Americans. J Gerontol A Biol Sci Med Sci.

[8] Keevil V, Mazzuin Razali R, Chin AV (2013). Grip strength in a cohort of older medical inpatients in Malaysia: a pilot study to describe the range, determinants and association with length of hospital stay. Arch Gerontol Geriatr.

[9] Cooper R, Kuh D, Hardy R (2010). Objectively measured physical capability levels and mortality: systematic review and meta-analysis. BMJ.

[10] Ekstrand E, Lexell J, Brogårdh C. Grip strength is a representative measure of muscle weakness in the upper extremity after stroke. Top Stroke Rehabil. 2016. 10.1080/10749357.2016.1168591. 10.1080/10749357.2016.1168591 27145212

[11] Cronin J, Lawton T, Harris N, Kilding A, McMaster DT. A brief review of handgrip strength and sport performance. J Strength Cond Res. 2017. 10.1519/JSC.0000000000002149. 10.1519/JSC.0000000000002149 28820854

[12] Bonitch-Góngora JG, Almeida F, Padial P, Bonitch-Domínguez JG, Feriche B (2013). Maximal isometric handgrip strength and endurance differences between elite and non-elite young judo athletes. Sci Mar Arts.

[13] Fry AC, Ciroslan D, Fry MD, LeRoux CD, Schilling BK, Chiu LZ. Anthropometric and performance variables discriminating elite American junior men weightlifters. J Strength Cond Res. 2006. 10.1519/R-18355.1. 10.1519/R-18355.1 17194241

[14] Garcia Pallares J, Lopez-Gullon JM, Torres-Bonete MD, Izquierdo M. Physical fitness factors to predict female Olympic wrestling performance and sex differences. J Strength Cond Res. 2012. 10.1519/JSC.0b013e31824741e7. 10.1519/JSC.0b013e31824741e7 22207259

[15] Grant S, Hasler T, Davies C, Aitchison TC, Wilson J, Whittaker A. A comparison of the anthropometric, strength, endurance and flexibility characteristics of female elite and recreational climbers and non-climbers. J Sports Sci. 2001. 10.1080/026404101750238953. 10.1080/026404101750238953 11461053

[16] Peterson BJ, Fitzgerald JS, Dietz CC, Ziegler KS, Ingraham SJ, Baker SE, et al. Division I hockey players generate more power than division III players during on- and off-ice performance tests. J Strength Cond Res. 2015. 10.1519/JSC.0000000000000754. 10.1519/JSC.0000000000000754 25436625

[17] Demirkan E, Koz M, Kutlu M, Favre M. Comparison of physical and physiological profiles in elite and amateur young wrestlers. J Strength Cond Res. 2015. 10.1519/JSC.0000000000000833. 10.1519/JSC.0000000000000833 25559900

[18] Massuca LM, Fragoso I, Teles J. Attributes of top elite team-handball players. J Strength Cond Res. 2014. 10.1519/JSC.0b013e318295d50e. 10.1519/JSC.0b013e318295d50e 23591948

[19] Pion JA, Fransen J, Deprez DN, Segers VI, Vaeyens R, Philippaerts RM, et al. Stature and jumping height are required in female volleyball, but motor coordination is a key factor for future elite success. J Strength Cond Res. 2015. 10.1519/JSC.0000000000000778. 10.1519/JSC.0000000000000778 25436627

[20] Sharma A, Tripathi V, Koley S. Correlations of anthropometric characteristics with physical fitness tests in Indian professional hockey players. J Hum Sport Ex. 2012. 10.4100/jhse.2012.73.09.

[21] Lo VE, Chiu Y-C, Tu H-H. Can we use grip strength to predict other types of hand exertions? An example of manufacturing industry workers. Int J Environ Res Public Health. 2021. 10.3390/ijerph18030856. 10.3390/ijerph18030856 PMC7908096 33498242

[22] Copay AG, Charles MT. The influence of grip strength on handgun marksmanship in basic law enforcement training. Policing. 2001. 10.1108/13639510110382241.

[23] Anderson GS, Plecas DB. Predicting shooting scores from physical performance data. Policing. 2000. 10.1108/13639510010355611.

[24] Kayihan G, ErsÖz G, Özkan A, Mitat K. Relationship between efficiency of pistol shooting and selected physical-physiological parameters of police. Policing. 2013. 10.1108/PIJPSM-03-2013-0034.

[25] Rodd D, Leasure-Woodburn M, Wilson G (2010). The effects of grip strength and firearm discharge. Indiana Law Enforc J.

[26] Kamarul T, Ahmad TS, Loh WYC (2006). Hand grip strength in the adult Malaysian population. J Orthop Surg (Hong Kong).

[27] Rice VJ, Williamson TL, Sharp M, Kumar S (1998). Using anthropometry and strength values to predict grip strength. Adv occup ergonom safety.

[28] Nicolay CW, Walker AL (2005). Surface EMG and anatomical measurements as predictors of grip strength. Int J Ind Ergon.

[29] Hossain MG, Zyroul R, Pereira BP, Kamarul T. Multiple regression analysis of factors influencing dominant hand grip strength in an adult Malaysian population. J Hand Surg Eur Vol. 2012. 10.1177/1753193411414639. 10.1177/1753193411414639 21816889

[30] Bhat AK, Jindal R, Acharya AM. The influence of ethnic differences based on upper limb anthropometry on grip and pinch strength. J Clin Orthop Trauma. 2021. 10.1016/j.jcot.2021.101504. 10.1016/j.jcot.2021.101504 PMC8321953 34367910

[31] Charles LE, Burchfiel CM, Fekedulegn D, Kashon ML, Ross GW, Sanderson WT, et al. Occupational and other risk factors for handgrip strength: the Honolulu-Asia Aging Study. Occup Environ Med. 2006. 10.1136/oem.2006.027813. 10.1136/oem.2006.027813 PMC2078007 16912086

[32] Orr R, Pope R, Stierli M, Hinton B. Grip strength and its relationship to police recruit task performance and injury risk: a retrospective cohort study. Int J Environ Res Public Health. 2017. 10.3390/ijerph14080941. 10.3390/ijerph14080941 PMC5580643 28825688

[33] Abe T, Thiebaud RS, Loenneke JP. Age-related change in handgrip strength in men and women: is muscle quality a contributing factor? Age (Dordr). 2016. 10.1007/s11357-016-9891-4. 10.1007/s11357-016-9891-4 PMC5005880 26874950

[34] Frederiksen H, Hjelmborg J, Mortensen J, McGue M, Vaupel JW, Christensen K. Age trajectories of grip strength: cross-sectional and longitudinal data among 8,342 Danes aged 46 to 102. Ann Epidemiol. 2006. 10.1016/j.annepidem.2005.10.006. 10.1016/j.annepidem.2005.10.006 16406245

[35] Vasquez E, Quiñones AR, Gensburg L (2019). Racial and ethnic differences between grip strength and functional limitations: results from NHATS 2010-2014. Innov Aging.

[36] Thorpe RJ, Simonsick E, Zonderman A, Evans MK. Association between race, household income and grip strength in middle- and older-aged adults. Ethn Dis. 2016. 10.18865/ed.26.4.493. 10.18865/ed.26.4.493 PMC5072478 27773976

[37] Mondol MK, Jana TK, Das J, Bishwas S (2009). Use of length of ulna for estimation ofstature in living adult male in Burdwan district and adjacent area of West Bengal. J Anat Soc India.

[38] Pugh-Kitingan J (2015). Cultural and religious diversity in Sabah and relationships with surrounding areas. Malay civilization.

[39] Jabatan Perangkaan Malaysia (2018). Buku Maklumat Perangkaan Malaysia.

[40] Jenkins DG, Quintana-Ascencio PF. A solution to minimum sample size for regressions. PLoS One. 2020. 10.1371/journal.pone.0229345. 10.1371/journal.pone.0229345 PMC7034864 32084211

[41] Díaz-Muñoz GA, Calvera-Millán SJ. Comparing the Camry dynamometer to the Jamar dynamometer for use in healthy Colombian adults. Rev Salud Bosque. 2019. 10.18270/rsb.v9i2.2794.

[42] Sanli SG, Kizilkanat ED, Boyan N, Ozsahin ET, Bozkir MG, Soames R, et al. Stature estimation based on hand length and foot length. Clin Anat. 2005. 10.1002/ca.20146. 10.1002/ca.20146 16187319

[43] Sharifi-Mollayousefi A, Yazdchi-Marandi M, Ayramlou H, Heidari P, Salavati A, Zarrintan S (2008). Assessment of body mass index and hand anthropometric measurements as independent risk factors for carpal tunnel syndrome. Folia Morphol (Warsz).

[44] Rhiu I, Kim W. Estimation of stature from finger and phalange lengths in a Korean adolescent. J Physiol Anthropol. 2019. 10.1186/s40101-019-0206-1. 10.1186/s40101-019-0206-1 PMC6805475 31640812

[45] Ruiz JR, Romero VE, Ortega FB, Sjöström M, Castillo MJ, Gutierrez A. Hand span influences optimal grip span in male and female teenagers. J Hand Surg Am. 2006. 10.1016/j.jhsa.2006.06.014. 10.1016/j.jhsa.2006.06.014 17027801

[46] Mathiowetz V, Weber K, Volland G, Kashman N. Reliability and validity of grip and pinch strength evaluations. J Hand Surg Am. 1984. 10.1016/s0363-5023(84)80146-x. 10.1016/s0363-5023(84)80146-x 6715829

[47] Coldham F, Lewis J, Lee H. The reliability of one vs. three grip trials in symptomatic and asymptomatic subjects. J Hand Ther. 2006. 10.1197/j.jht.2006.04.002. 10.1197/j.jht.2006.04.002 16861131

[48] Jäkel B, Kedor C, Grabowski P, Wittke K, Thiel S, Scherbakov N, et al. Hand grip strength and fatigability: correlation with clinical parameters and diagnostic suitability in ME/CFS. J Transl Med. 2021. 10.1186/s12967-021-02774-w. 10.1186/s12967-021-02774-w PMC8056497 33874961

[49] Armstrong CA, Oldham JA. A comparison of dominant and non-dominant hand strengths. J Hand Surg Br. 1999. 10.1054/jhsb.1999.0236. 10.1054/jhsb.1999.0236 10473148

[50] Sullivan M (2017). Statistics: informed decisions using data.

[51] Looney SW, Gulledge TR Jr. Use of the correlation coefficient with normal probability plots. Am Stat. 1985;39(1):75–9. 10.2307/2683917.

[52] Schober P, Boer C, Schwarte LA. Correlation coefficients: appropriate use and interpretation. Anesth Analg. 2018. 10.1213/ANE.0000000000002864. 10.1213/ANE.0000000000002864 29481436

[53] Pourhoseingholi MA, Baghestani AR, Vahedi M (2012). How to control confounding effects by statistical analysis. Gastroenterol Hepatol Bed Bench.

[54] Mitsionis G, Pakos EE, Stafilas KS, Paschos N, Papakostas T, Beris AE. Normative data on hand grip strength in a Greek adult population. Int Orthop. 2009. 10.1007/s00264-008-0551-x. 10.1007/s00264-008-0551-x PMC2903114 18414855

[55] Lim SH, Kim YH, Lee JS. Normative data on grip strength in a population-based study with adjusting confounding factors: Sixth Korea National Health and Nutrition Examination Survey (2014-2015). Int J Environ Res Public Health. 2019. 10.3390/ijerph16122235. 10.3390/ijerph16122235 PMC6616518 31242569

[56] Hatin WI, Nur-Shafawati AR, Zahri MK, Xu S, Jin L, Tan SG, et al. Population genetic structure of peninsular Malaysia Malay sub-ethnic groups. PLoS One. 2011. 10.1371/journal.pone.0018312. 10.1371/journal.pone.0018312 PMC3071720 21483678

[57] Tan CB, Ember M, Ember CR, Skoggard I (2005). Chinese in Malaysia. Encyclopedia of diasporas.

[58] Li K, Hewson DJ, Duchêne J, Hogrel JY. Predicting maximal grip strength using hand circumference. Man Ther. 2010. 10.1016/j.math.2010.06.010. 10.1016/j.math.2010.06.010 20708427

[59] Leyk D, Gorges W, Ridder D, Wunderlich M, Rüther T, Sievert A, et al. Hand-grip strength of young men, women and highly trained female athletes. Eur J Appl Physiol. 2007. 10.1007/s00421-006-0351-1. 10.1007/s00421-006-0351-1 17186303

[60] Koley S, Kumaar S. Correlations of handgrip strength with selected hand-anthropometric variables in university softball players. Biomed Hum Kinet. 2011. 10.2478/v10101-011-0020-7.

[61] Asha MT, Akter S, Tabassum R, Rahaman MS, Reza-Ul-Haq KM, Ara S (2020). A study to find out the correlation between handgrip strength and hand breadth of Bangladeshi male cricket batsman. Mymensingh Med J.

[62] Moy F-M, Azlan D, Noran NH. Predictors of handgrip strength among adults of a rural community in Malaysia. Asia Pac J Public Health. 2015:176–84. 10.1177/1010539513510555. 10.1177/1010539513510555 24285778

